# Assessing spring-mass similarity in elite and recreational runners

**DOI:** 10.3389/fphys.2023.1224459

**Published:** 2023-09-01

**Authors:** Geoffrey T. Burns, Nicholas Tam, Jordan Santos-Concejero, Ross Tucker, Ronald F. Zernicke

**Affiliations:** ^1^ School of Kinesiology, University of Michigan, Ann Arbor, MI, United States; ^2^ Division for Exercise Science and Sports Medicine, Department of Human Biology, University of Cape Town, Cape Town, South Africa; ^3^ Department of Physiology, University of the Basque Country UPV/EHU, Leioa, Spain; ^4^ Department of Physical Education and Sport, University of the Basque Country UPV/EHU, Vitoria-Gasteiz, Spain; ^5^ World Rugby, Dublin, Ireland; ^6^ Department of Orthopaedic Surgery, University of Michigan, Ann Arbor, MI, United States; ^7^ Department of Biomedical Engineering, University of Michigan, Ann Arbor, MI, United States

**Keywords:** biomechanics, distance, running, gait, stiffness, footwear, Kenyan

## Abstract

The dynamic complexity and individualization of running biomechanics has challenged the development of objective and comparative gait measures. Here, we present and explore several novel biomechanical metrics for running that are informed by a canonical inter-species gait template–the spring-mass model. The measures assess running mechanics systemically against the template via quantifying characteristics of a runner’s kinetics relative to the energy-conserving elastic system–i.e., their “spring-mass similarity”. Applying these metrics in a retrospective cohort investigation, we studied the overground kinetics of two heterogenous populations of runners in two footwear conditions: elite and recreational athletes in shod and barefoot conditions. Across all measures and within foot strike types, the elite runners exhibited mechanics that were more similar to those of the ideally elastic spring-mass template. The elite runners had more symmetric bounces, less discrepancy (i.e., greater coordination) between horizontal and vertical kinetic changes, and better fit to a spring-mass vertical ground reaction force time series. Barefoot running elicited greater kinetic coordination in the recreational runners. At a faster speed, the elites further improved their similarity to the template. Overall, the more economical elite group exhibited greater likeness to the linearly elastic, energy-conserving spring-mass system than their recreational counterparts. This study introduces novel biomechanical measures related to performance in distance running. More broadly, it provides new, approachable metrics for systemic quantification of gait biomechanics in runners across all demographics. These metrics may be applied to assess a runner’s global biomechanical response to a variety of interventions, including training adaptations, rehabilitation programs, and footwear conditions.

## 1 Introduction

The description and quantification of “good” running form has challenged biomechanists, physiologists, coaches, and athletes alike for decades. In his seminal training text, Fred Wilt posited that every runner must necessarily have a unique form, due to individual differences in physical make-up, likening it to a fingerprint (Wilt, 1959). Subsequent biomechanical investigations into the determinants of running performance have corroborated this individualization (Williams and Cavanagh, 1987; Nummela et al., 2007), and it was later echoed in the description of a runner’s “preferred movement path” (Nigg et al., 2015). Several reviews have synthesized the numerous investigations of the biomechanical aspects of running economy (a critical determinant of performance), and they have consistently identified several contributing factors such as lower vertical oscillation and greater leg stiffnesses (Anderson, 1996; Saunders et al., 2004; Moore, 2016). However, component-level kinematic determinants of performance and proper “form” have remained largely elusive (Moore, 2016). This may be explained by the nature of gait as a continuous, dynamic coordination of the myriad limb and joint segments (Hamill et al., 1999). When further considering the subsequent determinants of those motions (e.g., metabolic, neurologic, and biologic), the complexity approaches that of the “fingerprint” described by Wilt.

**FIGURE 1:**
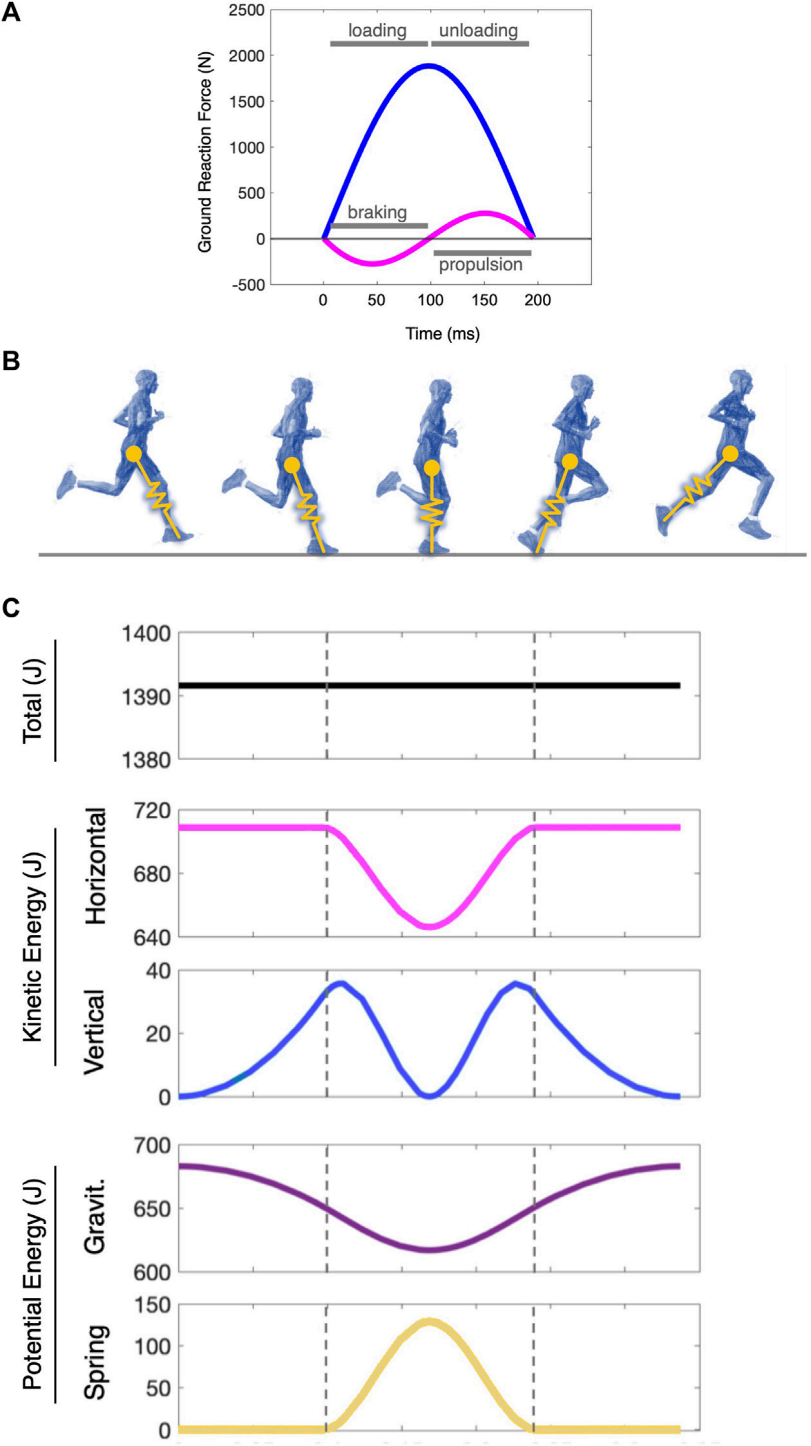
The mechanical energetics of the spring-mass model in running. **(A)** Ground reaction forces of the spring mass system with its vertical (blue) and horizontal (magenta) components. At midstance, the body transitions from loading to unloading and from braking to propulsion as its vertical and forward kinetic energies reach a minimum. In this ideal elastic spring-mass system, these transitions occur in phase, and with a symmetric bounce, the time of each segment is equal. **(B)** The interpretation of the spring-mass model in a human runner. **(C)** The mechanical energetics of a two-dimensional spring-loaded inverted pendulum (a “spring-mass” system) through one step cycle. The dashed lines indicate touchdown and takeoff. The blue lines denote the kinetic energy fluctuations broken out into the vertical and horizontal planes, and the purple lines indicate the potential energy fluctuations via gravitational forces and spring mechanics. The model is a 70 kg system with a 1.0 m leg moving at 4.5 m/s. Note: Energy itself does not have directionality (e.g., forward vs vertical), but it is vectorized here by the horizontal and vertical velocity progressions of the mass to illustrate those two-dimensional dynamics simultaneously as the step cycle progresses (Cavagna, 1975).

An alternative approach to that component-level kinematic characterization of gait is to assess it systemically, and the simplest system that captures the fundamental dynamics of running is that of the spring-loaded inverted pendulum—a spring-mass system (Blickhan, 1989; McMahon and Cheng, 1990). This two-dimensional model treats the runner as a simple point-mass on a linearly elastic “leg” spring that attacks and leaves the ground with a given touchdown angle. The model assumes ideal elasticity, and its dynamics are thus symmetric and energy-conserving through stance and flight. Its forward and vertical momentums change in-phase, with a transition from braking to propulsion occurring at mid-stance, which is the same instant the vertical position is at a minimum and the mass is transitioning from loading to unloading (Figure 1). This system has been proposed as the template that underlies running dynamics across species (Full and Koditschek, 1999; Full et al., 2000; Müller et al., 2016).

The aforementioned factors that have explained running economy (e.g., vertical oscillation and leg stiffness) are indeed systemic, “global” characteristics related to these spring-mass dynamics, so a comparison of runners to this template may be insightful for assessing gait behavior. However, given the complexity of the underlying dynamics (i.e., no analytical solution to its equations of motion exists (Schwind and Koditschek, 2000)), the means by which we can directly compare runners to this system is limited. Ahn and others used a metric termed the “percent congruity”–the proportion of time within a step cycle that kinetic and potential energies fluctuated in phase–to distinguish between pendular running (100%) and walking (0%) gaits in running frogs (Ahn et al., 2004). In this vein, a simple method for assessing similarity to the elastic spring-mass system was proposed by Cavagna and Legramandi, where they calculated the “similarity to a symmetric bounce” of a runner as the average of the ratios of the maximal upward and downward velocities of the center-of-mass and corresponding deceleration and acceleration times, both of which are 1.0 in an ideal spring-mass system (Cavagna and Legramandi, 2015). Their observations in ageing populations and in runners across speeds suggested that systemic spring-mass similarity may be discriminatory and indicative of underlying musculotendinous phenomena (Cavagna et al., 2008; Legramandi et al., 2013; Cavagna and Legramandi, 2015). However, the concept of “spring-mass similarity” has yet to be expanded upon or applied across other heterogenous groups or conditions. We undertook this investigation to explore the concept of “spring-mass similarity” and spring-mass behavior as an assessment strategy for systemic gait characteristics in runners of differing performance capacities and footwear conditions.

Elite distance runners are an appealing group in which to explore whether similarity or deviations from simple elastic systems (i.e., spring-mass behavior) differs between distinct populations or abilities, as the mechanical etiology of their high performance capacities and running economies have been suggested to be systemic in nature–i.e., multi-factorial interactions of a large number of variables (Cavanagh et al., 1977; Williams and Cavanagh, 1987; Burns et al., 2021a). Kenyan distance runners may be particularly enlightening to this end, as their performance capacities are uniquely prodigious (Tucker et al., 2015), and previous observations of their gait characteristics and musculotendinous behavior have suggested that they exploit elastic biomechanical mechanisms to efficiently store and return energy through stance and flight (Sano et al., 2013; Kunimasa et al., 2014; Sano et al., 2015; Santos-Concejero et al., 2017). We therefore set out to use this population of elite runners to explore the sensitivity and efficacy of several new metrics of spring-mass similarity. We hypothesized that these elite-level runners would exhibit greater similarity to the ideal elastic spring-mass system compared to a cohort of recreational runners. Furthermore, we hypothesized that these differences between the cohorts would be apparent in both shod and barefoot conditions, but that the changes between footwear conditions would be less distinct in the elite Kenyans, where familiarization with barefoot activity is common in childhood and adolescence (Lieberman et al., 2015; Aibast et al., 2017). Finally, we hypothesized that group differences would persist after controlling for foot strike pattern, for which we would anticipate rear foot striking to decrease similarity (Bobbert et al., 1991).

## 2 Materials and methods

### 2.1 Spring-mass similarity metrics

We explored three new metrics of spring-mass similarity: 1) the timing difference between the transition from braking to propulsion in the horizontal plane and the transition from loading to unloading in the vertical plane–the horizontal-vertical force timing difference (HV_TD_), 2) the time-normalized ratios of these events–the horizontal braking-to-propulsion and vertical loading-to-unloading ratios (HV_RATIO_), and 3) the overall similarity of a best-fit spring-mass vertical ground reaction force to that of a runner’s observed force as estimated by nonlinear regression (NLR)–the spring-mass fit (FIT_SM_). These metrics and their calculations are further described in Table 1. The first two metrics are an indication of the degree to which a runner’s forward and vertical contributions to their overall kinetic energy are coordinated and occur in phase, and the last is an assessment of the overall shape of the vGRF curve against the spring-mass template. Finally, we also used the aforementioned “similarity to a symmetric bounce” (SB) as an indication of the intra-stance kinetic symmetry (Cavagna and Legramandi, 2015).

**TABLE 1:** Spring-mass similarity metrics.

Metric	Name	Description	Calculation	Determinants
SB	Similarity to a Symmetric Bounce (Cavagna and Legramandi, 2015)	Average of the ratios of the maximal upward and downward COM velocities and the COM deceleration and acceleration timesBoth are 1.0 in a spring-mass system	$\frac{1}{2}\left(\frac{t_{dec.}}{t_{acc.}}+\frac{v_{down.}}{v_{up}}\right)\times 100\%$	t_dec._: deceleration time of COMt_acc._: acceleration time of COMv_down._: peak downward COM velocityv_up._: peak upward COM velocity
HV_TD_	Horizontal-Vertical Force Timing Difference	The difference between the time of transition from braking to propulsion in the horizontal plane and the time of transition from loading to unloading in the vertical plane. An indicator of kinetic coordinationEqual to 0 in a spring mass system	$t_{braking}-t_{loading}$	t_braking._: braking time in hGRFt_loading_: loading time in vGRF
HV_RATIO_	Horizontal-Vertical Force Timing Ratio	The contact time-normalized difference between the horizontal and vertical transition times. Also an indicator of kinetic coordination that is robust to differing contact timesEqual to 1.0 in a spring mass system	$\frac{t_{braking}/t_{propulsion}}{t_{loading}/t_{unloading}}$	t_braking._: braking time in hGRFt_propulsion_: propulsion time in hGRFt_loading_: loading time in hGRFt_unloading_: unloading time in hGRF
FIT_SM_	Spring-Mass vGRF Fit	Root-mean-squared error (RMSE) of best-fit spring-mass vGRF to subject’s observed vGRF	Nonlinear regression with subject’s observed vGRF time series (Burns et al., 2021b)	Subject’s observed vGRF time series
		Equal to 0 in a spring mass system		

COM: center-of-mass; hGRF: horizontal ground reaction force; vGRF: vertical ground reaction force.

### 2.2 Data collection

To explore these measures, we collated data collected from two previous investigations following similar experimental protocols. In the first, 25 male professional Kenyan runners were recruited via their management agency (10 km best: <28.9 min or a 5 km/half marathon equivalent) (Santos-Concejero et al., 2017). Ten ultimately declined to participate due to injury or competition conflict. From the second, 26 local male recreational runners were included from the cohort who completed overground trials at 12 kph during their visit (10 km best: <50 min; >4 h of training per week) (Tam et al., 2016). At the time of the studies, all participants were free of lower limb injury and refrained from hard training in the 2 days prior to the session. All participants provided written informed consent, and the studies were approved by the local ethics review board in accordance with the principles set forth in the Declaration of Helsinki.

For the kinetic assessments, both cohorts of participants ran in shod and barefoot conditions in randomized order at 12 km/h on a 40-m synthetic indoor track. Speeds were verified with photoelectric timing cells. The shod condition was performed in the subject’s habitual training shoe. Ground reaction forces were recorded from two 900 × 600 mm embedded force platforms (AMTI, Watertown, MA, United States) and sampled at 1,000 Hz. Five successful trials were captured for each subject in each condition, where a successful trial was defined as the participant running within ±5% of the target speed, striking the force plate with his right foot, and giving no indication of platform targeting. Additionally, the elite cohort completed 3 successful trials in each condition at 20 km/h.

For the metabolic assessments, subjects in both cohorts ran on a treadmill (h/p/cosmos Saturn, Nussdorf-Traunstein, Germany) set at a 1% gradient at 12 km/h for 6 min. Respiratory gas exchange was captured with an automated breath-by-breath metabolic measurement system (Cosmed Quark CPET, Rome, Italy). The system was calibrated prior to each trial. The flow rate calibration was performed using a 3-L syringe, and the gas exchange calibration was performed with reference gas (16% O2, 5% CO2) as well as ambient air per the manufacturer’s instructions. All tests were conducted under similar environmental conditions (22C and 50% relative humidity at 130 m altitude).

### 2.3 Data processing

The GRF recordings were filtered using a low-pass, fourth order Butterworth filter with a cutoff frequency of 60 Hz and a landing/take-off threshold of 50 N. Subjects were classified as rearfoot striking (RF) or non-rearfoot striking (NRF) based on the presence of an impact peak in the vGRF in the shod condition. Spring-mass similarity was quantified using the method of Cavagna and Legramandi, where they calculated the “similarity to an elastic bounce” (SB) as the average of the ratio of the maximal downward velocity (v_down_) and upward velocity (v_up_) of the COM and the ratio of the deceleration time (t_dec_) to acceleration time (t_acc_) of the COM during stance (Legramandi et al., 2013; Cavagna and Legramandi, 2015). The forward and vertical velocities were determined by integrating the respective ground reaction forces in accordance with the method presented by Cavanga (Cavagna, 1975). Here, the average horizontal velocity during the step cycle is taken as the forward running speed and the average vertical velocity through the step cycle is assumed to be zero, allowing the calculations of the integration constants that satisfy those conditions. From these center-of-mass velocity time series, v_down_, v_up_, t_dec_, and t_acc_ can be calculated (Legramandi et al., 2013; Cavagna and Legramandi, 2015).

This spring-mass similarity was further quantified with three additional metrics (Table 1). First, the difference between the time at which the horizontal GRF crossed 0 N, indicating a transition from braking to propulsion in the horizontal plane, and the time at which the vertical GRF reached its peak, indicating a transition from loading to unloading in the vertical plane, was measured. In a perfectly elastic bounce, this horizontal and vertical timing difference (HV_TD_) would be zero. Second, the ratio of the horizontal braking and propulsion times to the vertical loading and unloading times was calculated. The ratio of the braking to propulsion ratio and the loading to unloading ratio (HV_RATIO_) is conceptually similar to the HV_TD_ in that it attains unity (1.0) in an elastic system, but normalizes any asymmetry to the asymmetry in the loading and unloading phases of stance and to the total contact time of the stance. Third, a best-fit spring-mass vGRF was modeled for each step of each runner using nonlinear regression (Burns et al., 2021b). The root mean-squared error (RMSE) of this best-fit spring-mass vGRF against the observed vGRF was recorded as a measure of the vGRF similarity to an ideal spring-mass system (FIT_SM_), where a lower value indicates better fit.

The nonlinear regression (NLR) method for the FIT_SM_ metric uses a spring-mass vGRF time series function (Equation 1) that is defined by four parameters: stiffness (k), touchdown angle (α_TD_), leg length (L_0_), and contact time (t_c_). The technique determines the four parameters that minimize the error between the observed vGRF time series and the modeled spring-mass vGRF time series. In doing so, it accurately models spring-mass dynamics while being liberated from the traditional assumptions used in spring-mass calculations (Burns et al., 2021b). Here, the stochastic expectation maximization algorithm was used to fit model parameters (Feodor Nielsen, 2000), and the models for each subject were initiated with values that minimized the sum of squared errors for their aggregate steps via nonlinear least-squares optimization. Bounds were set at a lower limit of 5 kN/m, 63°, 80 cm, and an upper limit of 0.12 s and 30 kN/m, 74.5°, 120 cm, and 0.40 s for the four spring-mass parameters, respectively. Mixed-effect models were fit for each subject-condition with the steps within conditions modeled with random effects. The vGRF data were downsampled to 500 Hz to facilitate convergence. All fitting was performed using the Nonlinear Regression toolbox in MatLab (2019a, MathWorks, Natick, MA, United States). For further details and software code pertaining the NLR method and implementation, see Burns et al. (Burns et al., 2021b).

$$F_{y}\left(t\right)=\left(k\frac{L_{0}-\frac{g}{8}t_{c}-L_{0}\sin\alpha_{TD}}{1-\frac{k}{m}{\left(\frac{t_{c}}{\pi }\right)}^{2}}\right)\sin\left(t\frac{t_{c}}{\pi }\right)\left(1-\frac{1}{1+e^{{-10}^{10}\left(t-t_{c}\right)}}\right)$$ (1)

### 2.4 Data analysis

The analyses of the spring-mass similarity measures (SB, HV_TD_, HV_RATIO_, and FIT_SM_) were conducted using mixed-effect model linear regression, treating cohort, shoe condition, foot strike, and speed as fixed effects with interactions on cohort and shoe condition. Each subject was assigned a random effect intercept. For the linear mixed-effect models, the fixed effects were assessed for significance via Satterthwaite’s method. Statistical test criterion in all models used a Type I error control of α < 0.05. MatLab (2019a, MathWorks, Natick, MA, United States) was used for all data processing and NLR modeling, and R (v4.0.4, R Foundation for Statistical Computing, Vienna, Austria) was used for all statistical analyses.

## 3 Results

The elite cohort in our study were more energetically efficient at 12 km/h than their recreational counterparts (running economies: 192.6 ± 11.2 *versus* 218.9 ± 15.9 mL O_2_/kg/km as mean ± std. dev.) and markedly faster over 10 km (10 km bests: 28:43 ± 0:22 *versus* 43:33 ± 5:10). The aggregate subject characteristics from each cohort are provided in Table 2, and the analysis of the spring-mass similarity metrics are compiled in Table 3.

**TABLE 2:** Subject characteristics.

Characteristic	Elite	Recreational
Subjects (n)	15	26
Foot Strike (RF/NRF)	7/8	20/6
Age (yr)	23.7 ± 4.0	28.5 ± 5.3
Mass (kg)	54.9 ± 5.8	72.3 ± 10.8
Height (cm)	170.5 ± 6.1	175.9 ± 8.6
Running Economy (mL O_2_/kg/km)	192.6 ± 11.2	218.9 ± 15.2
10 km Best (min:sec)	28:42.9 ± 00:21.7*	43:36.7 ± 05:10.0

Data are provided as mean ± s.d. ^*^n = 13; RF: rearfoot; NRF: non-rearfoot.

**TABLE 3:** Analysis of spring-mass similarity metrics across cohorts and conditions. (A) Similarity to a symmetric bounce (SB). (B) The horizontal-vertical force timing difference (HV_TD_). (C) The horizontal-vertical force timing ratio (HV_RATIO_). (D) The spring-mass vGRF fit (*FIT_SM_*) given as the root-mean-squared error (RMSE) of the model fit, where a decrease in the value indicates better fit. Data are presented as the regression coefficients from the mixed-effects models where *Recreational* runners in the *Shod* condition with *RF* strike at *12 kph* are the intercept (i.e., baseline) model. As such, the top row in A-D indicates the mean for that cohort in those conditions. The independent contribution of each subsequent fixed effect (i.e., Elite runners, Barefoot (BF) condition, NRF strike, 20 kph, and the Elite x Barefoot (BF) interaction) are presented below that. E.g., across all conditions, Elite runners have a HV_TD_ that is 16.3−6.3 = 10.0 m, and in the BF condition they have an HV_TD_ that is 16.3−6.3−1.8 + 3.5 = 11.7 m. The standard error of the measure (sem) for each effect is provided, and the statistical significance of each effect is indicated as: ✻ *p* < 0.05, ✻✻ *p* < 0.01, and ✻✻✻ *p* < 0.001.

A	SB (%)	sem	p	sig.
Recreational	92.7	0.8	<0.001	
Elite	+2.7	1.3	0.048	✻
Barefoot	+0.3	0.5	0.557	
Elite x BF	+0.5	1.1	0.636	
NRF strike	−2.0	1.3	0.145	
20 km/h (Elite)	−5.8	0.8	<0.001	✻✻✻

B	HV_TD_ (ms)	sem	p	sig.
Recreational	16.3	1.6		
Elite	−6.3	2.6	0.018	✻
Barefoot	−1.8	0.6	0.003	✻✻
Elite x BF	+3.5	1.2	0.003	✻✻
NRF strike	−6.0	2.6	0.026	✻
20 km/h (Elite)	−8.9	0.9	<0.001	✻✻✻

C	HV_ratio_	sem	p	sig.
Recreational	1.32	0.03		
Elite	−0.11	0.05	0.047	✻
Barefoot	−0.03	0.01	0.048	✻
Elite x BF	+0.05	0.03	0.041	✻
NRF strike	−0.11	0.05	0.037	✻
20 km/h (Elite)	−0.20	0.02	<0.001	✻✻✻

D	FIT_SM_ (N)	sem	p	sig.
Recreational	160.6	8.6		
Elite	−37.7	14.3	0.012	✻
Barefoot	14.1	6.5	0.034	✻
Elite x BF	−41.7	13.9	0.005	✻✻
NRF strike	19.3	8.9	0.033	✻
20 km/h (Elite)	−27.6	13.0	0.038	✻

### 3.1 Elite distance runners were more similar to the simple elastic spring-mass system

**FIGURE 2:**
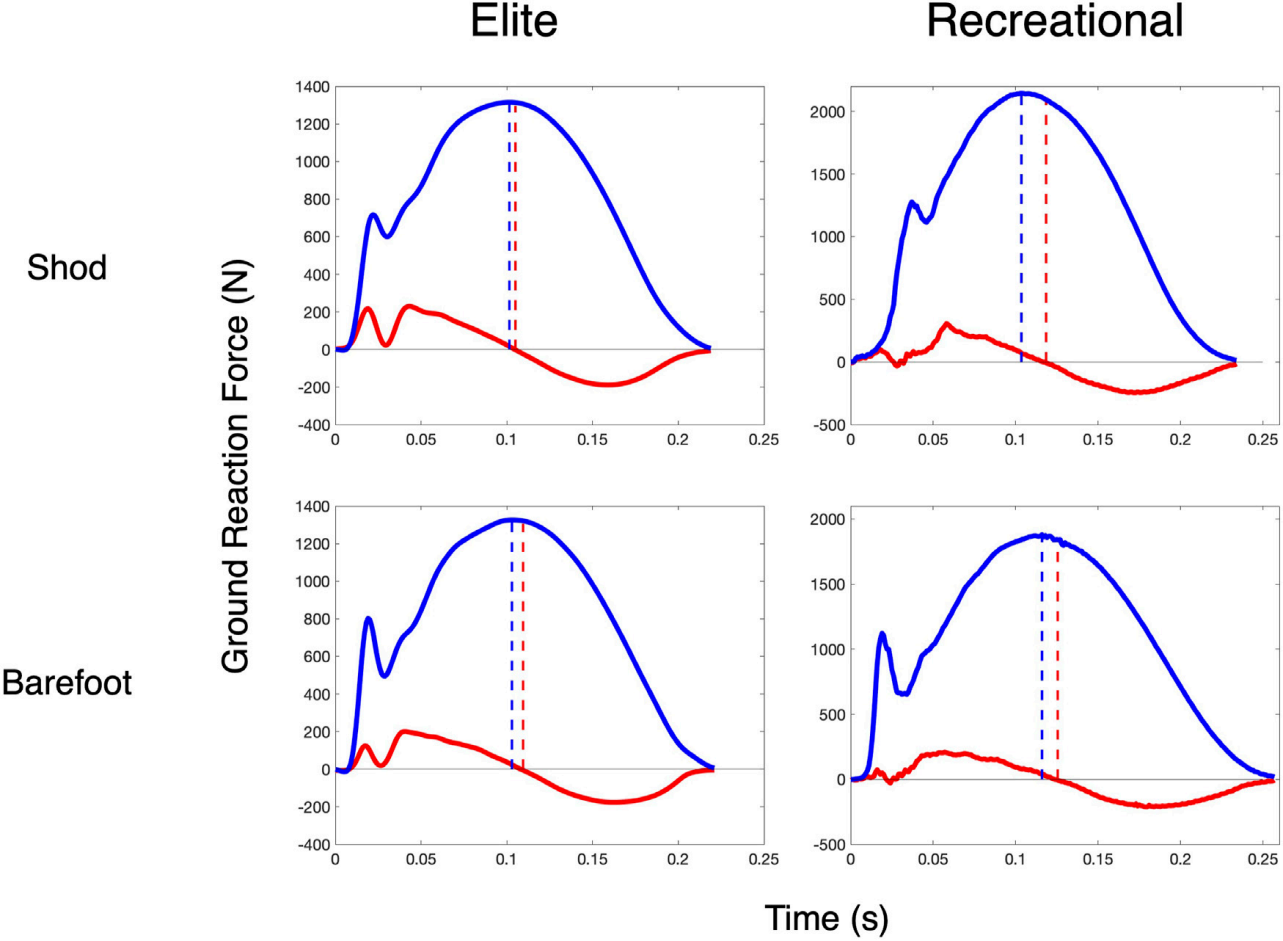
Ground reaction force timing differences. HV_TD_ in two rearfoot striking subjects in each shoe condition at 12 km/h. Blue solid lines are the observed vGRF, and blue dashed lines indicate the time it reaches its peak. Red solid lines are the observed hGRF, and the red solid lines indicate the time that the hGRF transitions from braking to propulsion.

Across all measures, the faster, more efficient elite runners were more similar to the spring-mass system than the recreational runners. Running at 12 km/h, their SB metric was 95.4% compared to 92.7% in the recreational runners (effect SEM: 1.3%). With respect to the GRF timing differences, their discrepancy was lower (i.e., horizontal and vertical kinetic progressions were more coordinated), with an HV_TD_ of 10.0 m vs 16.3 m (effect SEM: 2.6 m) and a normalized timing difference ratio (HV_RATIO_) of 1.21 vs 1.32 (effect SEM: 0.05). The data of two representative subjects are shown in Figure 2. Their overall spring-mass model “fit” as assessed by NLR (FIT_SM_) was better, with average model errors of 122.9 N compared to 160.6 N (effect SEM: 14.3 N) in the recreational runners. Four representative subjects’ data are shown in Figure 2.

### 3.2 Recreational runners improved kinetic coordination during stance when barefoot, but elite runners were unaffected

With respect to the effect of shoes, the HV_TD_ and HV_RATIO_ revealed distinct responses between the two cohorts. For the recreational runners, the HV_TD_ and HV_RATIO_ decreased in the barefoot condition (HV_TD_ change: –1.8 m, SEM: 0.6 m and HV_RATIO_ change: –0.03, SEM: 0.01), whereas they increased slightly in the elite runners (HV_TD_ change: +1.7 m, interaction SEM: 1.2 m and HV_RATIO_ change: +0.02, interaction SEM: 1.2). This change is illustrated in a representative subject from each cohort in Figure 2. The FIT_SM_ decreased in the barefoot condition for the recreational runners, with a 14.1 N increase in fit error, but it improved in the elite runners by 27.6 N (interaction SEM: 13.9). SB did not change within subjects between the two footwear conditions.

### 3.3 Spring-mass similarity in elite distance runners persisted for both rearfoot and non-rearfoot striking patterns

**FIGURE 3:**
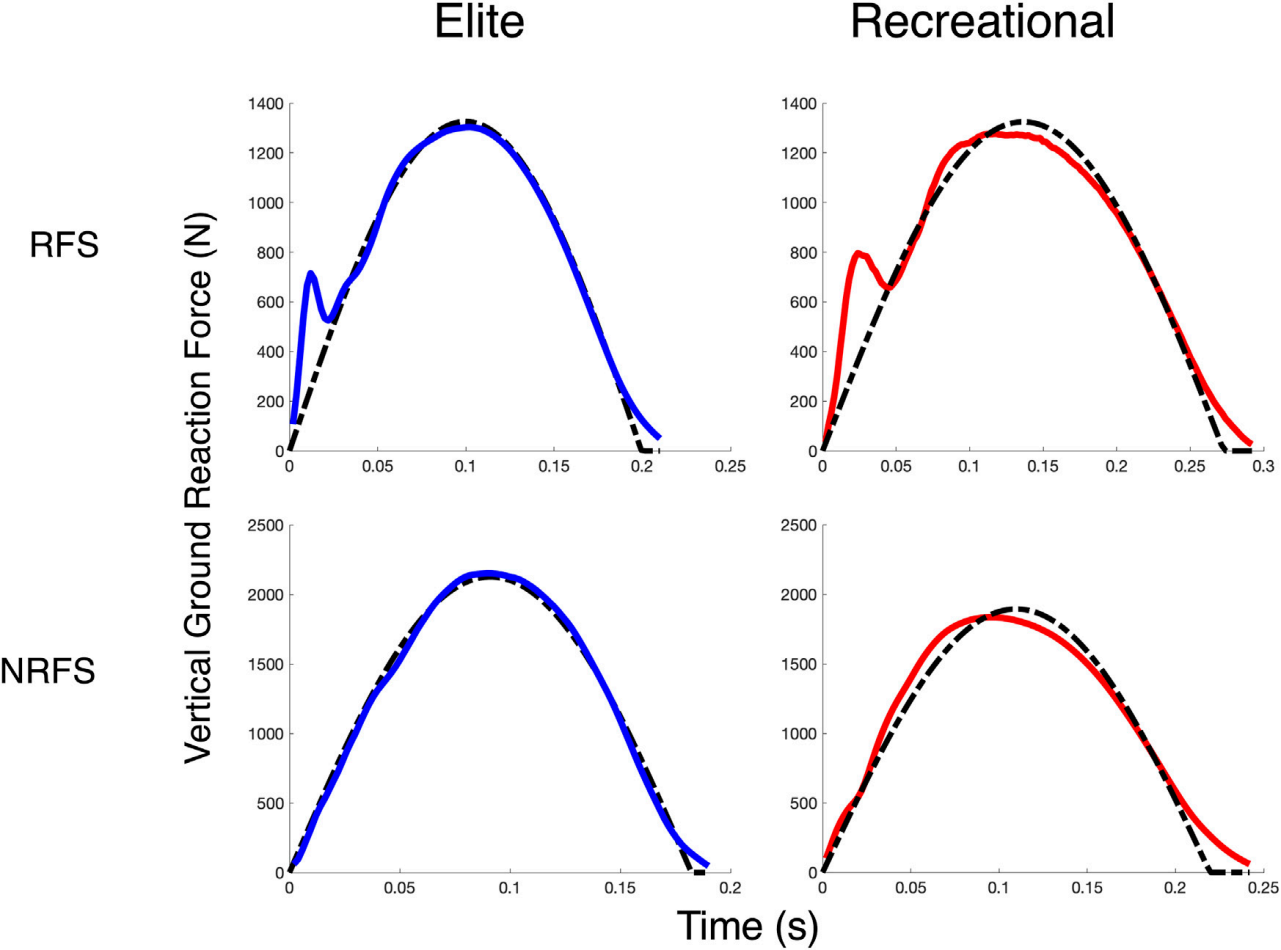
Spring-Mass Fits. FIT_SM_ for four subjects from each cohort and foot strike type in the shod condition at 12 km/h. The solid line (blue for elite runners, red for recreational runners) is the observed vGRF, and the dashed line is the best-fit spring mass vGRF estimated via NLR.

Foot strike type followed similar patterns, where RF striking runners demonstrated greater HV_TD_ than NRF striking runners (+6.0 m, effect SEM: 2.6 m) and enhanced HV_RATIO_ ratios (1.21 vs 1.32), effect SEM: 0.05). Similarly, the NRF striking runners demonstrated better overall fit to the SM model, with an error reduction of 19.3 N (effect SEM: 8.9). These fit characteristics are illustrated for four subjects in Figure 3. Foot strike type did not significantly influence SB.

### 3.4 Elite distance runners increased their similarity to spring-mass systems at faster speeds

**FIGURE 4:**
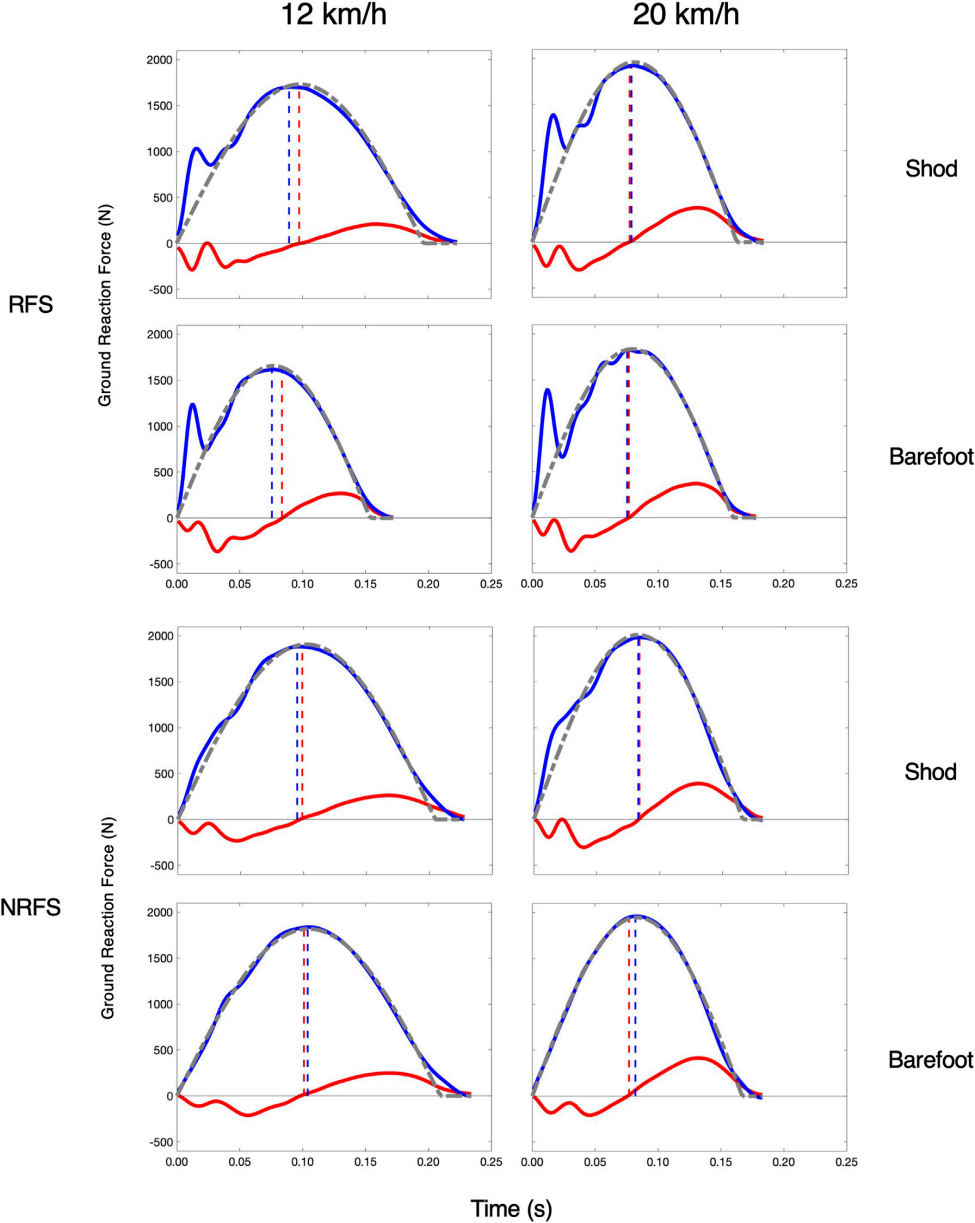
Spring-Mass Similarity at 12 km/h and 20 km/h in elite runners. One rearfoot striking (RFS) subject (top) subject and one non-rearfoot striking (NRFS) subject (bottom) at each speed and in each footwear condition. Blue (vGRF) and red (hGRF) dashed lines indicate the HV_TD_ and the gray dashed line is the FIT_SM_.

We also examined the similarity characteristics of the elite runners at 20 km/h. At this faster speed, their HV_TD_ approached zero (1.1 m; effect SEM: 0.9 m), and the HV_RATIO_ approached unity (1.01, effect SEM: 0.02). Their FIT_SM_ further improved from 122.9 N to 95.3 N (effect SEM: 13.0 N). Curiously, despite better kinetic coordination and vGRF similarity, they exhibited a decrease in their overall stance symmetry, with their SB decreasing by 5.9% (effect SEM: 0.8%). Figure 4 shows these patterns for two representative subjects. All model results are compiled in Table 3.

## 4 Discussion

We presented three new metrics to assess running gait against a spring-mass template: the time difference between kinetic transition events in the horizontal and vertical forces (HV_TD_), the time-normalized ratio of the braking-to-propulsion and loading-to-unloading events (HV_RATIO_), and the overall similarity of the runner’s vertical force to a best-fit spring-mass system (FIT_SM_). Using these measures and the “symmetric bounce” metric, we compared the elastic spring-mass behavior of elite distance runners to a cohort of recreational runners in shod and barefoot conditions. Overall, the metabolically efficient and high-performing elite distance runners had gaits that more closely mimicked the characteristics of an energy-conserving, ideally elastic spring-mass system, even after controlling for foot strike type.

### 4.1 Similarity of elite *versus* recreational runners

These results support our initial hypothesis that the high-performing distance runners would behave more like the simple spring-mass system. This difference was apparent across all metrics, but was most pronounced in the HV_TD_ and FIT_SM_ measures. Their braking-to-propulsion transition occurred 6.3 m closer to their vertical peak, which indicates greater coordination between their horizontal and vertical kinetic energy fluctuations. Here, the recreational cohort was continuing to do negative mechanical work in the horizontal plane–i.e., braking–after they had finished doing negative work in the vertical plane and had begun doing positive work (i.e., unloading). This kinetic discrepancy represented 6.7% of their total ground contact time compared to only 4.8% of the elites’ ground contact time (Figure 2). This improved coordination of kinetic energy through the gait cycle may contribute to the superior overall metabolic efficiency that they demonstrated relative to the recreational runners. Moore and others observed a similar phenomenon in a group of recreational runners, where better alignment of the leg axis and the resultant ground reaction force vector corresponded to improved economy (Moore et al., 2016). This alignment of the resultant GRF vector is thought to minimize the moments about each joint and reduce the overall muscle activity in the lower limbs, thereby improving locomotor efficiency (Chang et al., 2000). Here, better coordination of the two-dimensional Cartesian GRF components was also characteristic of the group with superior running economies and better running performances. Furthermore, their SB was also 2.7% more similar to an elastic system. This difference was the same magnitude as that observed by Cavagna and others between teenagers and adults (Legramandi et al., 2013). Furthermore, here, in comparing overall vGRF curves to spring-mass vGRFs, the elite runners had a model error that was 24% lower than the recreational runners, indicating that they coordinate their complexes of myriad body segments and muscle activations through the loading and unloading of stance to more closely follow the time course of a single point-mass on a pendular, linearly elastic spring. In the aggregate, these findings suggest that the elite runners control their global mechanical behavior to decelerate and accelerate their bodies in a smoother, more coordinated fashion.

The differences between the groups were still apparent after controlling for foot strike type. We anticipated that runners with a RF strike pattern would generally exhibit reduced absolute similarity, especially in the FIT_SM_ measure, due to the presence of the impact peak and greater initial vertical loading rates (Cavanagh and Lafortune, 1980; Lieberman et al., 2010). That hypothesis was supported, as across both groups, the NRF striking runners had a 29% lower model error in comparing their vGRF to that of a spring-mass system and a braking-to-propulsion transition that was 6 m closer the loading-to-unloading transition. While a greater proportion of the elite runners exhibited a NRF strike pattern (47% vs 27%), the effects were independently significant, where RF striking elite runners were more similar to the spring-mass system than their RF-striking recreational counterparts (e.g., Figure 3). This indicates that independent of foot strike type, the elite runners move through the stance phase with better kinetic coordination and spring-mass similarity. This also highlights the importance of assessing these metrics within individuals and within the context of foot strike characteristics. Foot strike type itself does not influence economy, and that changing it within an individual may be detrimental (Gruber et al., 2013). Here, it was further demonstrated that within a given strike pattern, runners vary in their similarity to idealized spring-mass mechanics, and that degree of similarity was related to differences in performance capacity.

These kinetic observations parallel the musculotendinous observations of Sano et al., who found more efficient elastic mechanics in elite Kenyan distance runners (Sano et al., 2013; Kunimasa et al., 2014; Sano et al., 2015). They observed that the Kenyan runners had longer Achilles tendon structures, and that in hopping, they exhibited lower overall stretching and shortening amplitudes and fascicle length changes of their medial gastrocnemius (MG) muscle, but greater stretching-shortening ratios during pre-activation and contact. These phenomena resulted in greater maximal hopping heights, which the investigators suggested was indicative of greater efficiency in elastic energy utilization (Sano et al., 2013). The same patterns were later observed in running, where a group of elite Kenyan runners had lower stretching and shortening amplitudes in the MG, but greater tendon contribution to the overall muscle-tendon unit’s shortening as compared to a group of similarly elite Japanese distance runners. Furthermore, they again exhibited lower pre-activation-to-braking muscle activity, facilitating greater isometric work of the MG during contact (Sano et al., 2015). Together, this indicated that their lower limbs were more efficiently transferring and recycling the kinetic energy from the flight phase to elastic strain energy in the contact phase. The lower activations of the MG and tibialis anterior in the braking phase may help explain our observations of better coordination of the horizontal braking phase to the start of vertical unloading. Lower muscle activity may prevent unnecessary extension of the braking phase or facilitate more controlled coordination of the forward and vertical center-of-mass decelerations. Müller et al. observed a similar phenomenon in subjects running over steps, where increasingly higher steps elcited increasingly lower MG activity, allowing the runners to maintain a more consistent center-of-mass trajectory through a perturbed stance (Müller et al., 2010). The decreased MG activity observed there may have served to mediate the potential disruption to the runners’ kinetic coordination, where their compensatory alterations to their global mechanics facilitated a better coupling of the otherwise perturbed braking and loading periods. Finally, Sano et al. also observed lower foot-lever-ratios in the elite Kenyan runners compared to their Japanese counterparts, a measure of the Achilles moment-arm to the length of the forefoot (Kunimasa et al., 2014). This may support the proposition of Maykranz and Seyfarth that a compliant ankle and the foot’s lever action contribute to takeoff-landing asymmetries and deviance from a symmetric bounce in running (Maykranz and Seyfarth, 2014), which was more characteristic of the recreational runners we observed. With these combined findings, one could hypothesize that longer foot levers may also cause greater deviation from elastic mechanics in the final moment of stance, perhaps further explaining some of the spring-mass similarity exhibited by the elite runners here.

### 4.2 Effect of footwear on spring-mass similarity

In both cohorts, the barefoot condition elicited mixed results with respect to the runners’ similarity to the spring-mass systems. The effects of running barefoot on the HV_TD_ and HV_RATIO_ metrics indicated a small but significant improvement in the coordination of the vertical and horizontal force components in the recreational runners, reducing the braking and loading discrepancy by 1.8 m, or 1% of their gait cycle. However, the cohort interaction was significant and opposite, indicating that the elite runners had a slight decrement in that timing difference, increasing it by 1.7 m, or 1% of their gait cycle. The time-normalized HV_RATIO_ ratio followed these patterns in both cohorts. This was unexpected, as we anticipated that the transition timings would be unaffected by the footwear condition (Divert et al., 2005). However, the greater initial loading rates common with habitually shod runners in a barefoot condition may explain the recreational runners’ shorter relative braking times (Tam et al., 2016), and the greater prevalence of habitual barefoot activity in childhood and adolescence in Western Kenya may have influenced the variable response in the elite cohort, as they were all born and raised in the Rift Valley region (Lieberman et al., 2015; Aibast et al., 2017). The SB was unaffected by the footwear condition. Legramandi and others observed the SB to decrease in runners as impact accelerations increased (as is common with barefoot running), but here, the changes may have been small enough, and perhaps offset by compensatory changes in upward velocity during toe-off so as not to substantially affect the overall symmetry measure (Legramandi et al., 2013). Finally, the FIT_SM_ decreased in the recreational runners by 9%, but improved in the elite runners by 11%. The reason for this deviation may be similar to that observed in the HV_TD_ patterns, where greater vertical impact transients associated with barefoot running in the habitually shod recreational cohort may have driven the decreasing model similarity, whereas barefoot familiarity in the elite Kenyan runners may have enhanced overall fit through stance (Lieberman et al., 2010; Tam et al., 2016). This complex footwear relation within the four spring-mass similarity metrics–e.g., enhanced HV_TD_ and HV_RATIO_, decreased FIT_SM_, and equivocal SB in the recreational runners–may provide some further insight as to why examinations of efficiency in shod and barefoot conditions have provided varying results, often with undetectable net differences (Divert et al., 2008; Franz et al., 2012).

### 4.3 Effect of speed on spring-mass similarity

Similarity to the spring-mass system increased at 20 km/h in the kinetic coordination and vGRF fit metrics in the elite runners (Figure 4). Cavagna observed that symmetry in the stance phase increased with speed, with braking and pushing times approaching unity above 14 km/h (Cavagna, 2006). He hypothesized this was due to greater isometric activity of the lower limb muscles at higher speeds, with greater tendinous contributions. Thus, a greater proportion of the mechanical work would be performed by the storage and release of elastic strain energy. Here, the HV_TD_ and HV_RATIO_ also approached perfect coordination, with a braking and loading difference of only 1.1 m and the ratios of 1.01 for the horizontal and vertical acceleration changes. Contrary to the findings of Cavagna and others (Cavagna et al., 2008; Cavagna and Legramandi, 2015), the SB decreased at the faster speeds. This was due to a greater pushing time relative to the braking time, which itself was likely an artifact of an extended ground contact at toe-off due compliant ankle mechanics (Maykranz and Seyfarth, 2014). That this contribution increased at the faster speed is not itself indicative of overall spring-mass mechanics, as it occurs in the final moments of stance when the body is nearly unloaded. Correspondingly, the overall fit of the runners’ vGRFs to the spring-mass system did improve substantially at the faster speed, which itself is a metric improved by symmetry in the vertical kinetics of the runner. Together, these observations support the idea that runners behave more similarly to simple spring-mass systems at faster speeds.

### 4.4 Differences between similarity metrics

This investigation assessed a runner’s similarity to the spring-mass system with several metrics that each characterize distinct components of the elastic system’s dynamics. The first metric, SB, is a measure of energetic symmetry within the stance phase (Cavagna and Legramandi, 2015). It is an average of the two common asymmetries identified by Cavanga, which are the timing of the braking and pushing (center-of-mass deceleration and acceleration, respectively) and maximal upward velocity in the latter phase of stance and the maximal downward velocity in the initial phase (Cavagna et al., 2008; Cavagna, 2009; Cavagna and Legramandi, 2015). These two values are equal in an ideal spring-mass system, as energy is conserved throughout the stance phase with perfectly elastic dynamics. We presented three additional new metrics here that move beyond the symmetry of the step to quantify the kinetic coordination and further describe the similarity of a runner to the simple system throughout stance. The first, the HV_TD_, is a measure of alignment of forward and vertical energy fluctuations, as these change perfectly in phase in a spring-mass system (Figure 1). A symmetric bounce does not necessarily demand perfect coordination in the vertical and horizontal force progressions (i.e., timing, relative phase shifts, and magnitudes of each component could theoretically offset each other for equal braking and pushing), nor does a lack of difference in the HV_TD_ imply a symmetric bounce. The HV_RATIO_ ratio normalizes these kinetic timings in each phase of stance to each other so that discrepancy between the braking and loading times is relative to its counterpart in the latter half of stance. We observed this metric to follow the same patterns of the HV_TD_, indicating that the trends we observed in the absolute timing differences were not due to contact time differences.

The final metric, the FIT_SM_, assesses the overall similarity of the runner’s vGRF shape to that of an ideal spring-mass model fit to their behavior via nonlinear regression. This characterizes the constancy of the loading and unloading progression, which is smooth and symmetric in a spring-mass system, as the linear spring is dynamically compressed and released. A perfect FIT_SM_ would indicate symmetry in the vGRF component, but it does not necessarily imply a perfect SB metric, as the horizontal braking and propulsion could be asymmetric. It is conceptually similar to a force-displacement curve restricted to the vertical plane, which would be a perfectly linear line in an ideal system, but it is less biased by large deviations in the early and later phases of stance. A force-displacement assessment is heavily influenced by these aspects, as the force magnitudes are low and the displacements comparatively large (Maykranz and Seyfarth, 2014; Cavagna and Legramandi, 2015). The FIT_SM_ is influenced by common characteristics of vGRF curves, such as impact transients and take-off asymmetries, but it distinguishes the “smoothness” of the force progression despite these characteristics, capturing the overall systemic rise and fall of the curve. Moreover, it is not constrained to the exact “observed” contact time (e.g., the discrepancy between the fitted and observed curve in the final moments of stance, as seen in Figure 3), which often may be biased near terminal stance by compliant ankle mechanics (Maykranz and Seyfarth, 2014). Rather, it captures the effective global spring-mass dynamics and more accurately models that system for the runner (Burns et al., 2021b). With this metric, we saw NRF striking runners exhibit better FIT_SM_ characteristics than RF striking runners, but within those RF striking runners, the method discriminated a better FIT_SM_ among the elite RF striking runners. These fit patterns demonstrate that within foot strike types, runners can behave more or less like spring-mass systems. Together, this collection of measures characterizes the kinetic symmetry, coordination, and consistency of a runner during stance against a spring-mass template.

### 4.5 Considerations and limitations

Several considerations are important in the interpretation of our findings. The first is the heterogeneity of the two groups. Because the cohorts were elite professional Kenyan runners of Kalenjin ethnicity and non-elite recreational South African runners of varied ethnicities, it is impossible to definitively ascribe the differences observed here to either the performance capacity or demographic, or indeed a further range of anthropometric and physiological differences between the groups. For example, the groups varied substantially in mass, which may have had inertial effects that underly the overserved differences in the spring-mass similarities. It is possible that the smaller moment of inertia of their COM about their center-of-pressure would enable better control and phasic coordination of their horizontal and vertical decelerations and transitions as they move through stance, resulting in the better demonstrated HV_TD_ values. As body mass and body mass index are linked to running performance (Bale et al., 1986; Herrmann et al., 2019), it would be difficult to examine these effects independently. Manipulating mass within individuals–either acutely or during a period of weight change–may be one way to further explore the effects of mass on the kinetic coordination measures presented here.

The elites themselves were also of a unique ethnic demographic in the landscape of global distance running: Kalenjin Kenyans. Would elite runners of a different ethnic background with differing upbringings and differing anthropometries exhibit these same similarity patterns? Sano and others observed distinct musculotendinous behavior in elite Kenyans when compared to similarly high-level Japanese runners, so there may be ethnic relations underpinning these observations. Moreover, the economy and performance homogeneity was high within each group, as was the heterogeneity between groups, preventing this measure from being used as a covariate to more distinctly model the effects. To facilitate future investigations and sample size estimations, individual data within each group are provided in Supplementary File S1.

Furthermore, the design of the current study aimed to characterize elastic behavior and spring-mass similarity in two distinct populations of runners so as to explore the sensitivity of the new metrics presented. As such, it came at the cost of confounding the effect attribution (e.g., anthropometry, ethnicity, *etc.*), but it does not compromise the conclusions that the more efficient, higher performing runners exhibited distinct spring-mass similarities. Elite runners themselves often present with myriad interacting demographic distinctions that facilitate their elevated performance potentials. Future investigations within elite populations or across a greater continuum of performance capacity would certainly reveal further insights into the mechanisms and relations of spring-mass behavior and performance observed here.

The *post hoc* nature of the investigation must also be taken into consideration. As the analysis was conducted on previously collected data (Tam et al., 2016; Santos-Concejero et al., 2017), we were limited by both sample size and design. The effects were sufficiently large for detection across the measures of interest, but it is unclear whether some of the unaffected factors, such as the conditions within the SB analysis, were truly indicative of no effect or the result of Type II error. We attempted to mitigate that by assessing repeated steps within subjects, but future investigations into these behaviors would benefit from using these findings to anticipate effect and sample sizes. Similarly, because the investigation was performed retrospectively, we did not have data on the recreational runners at the faster speeds. Given the interactions observed between cohorts and footwear conditions, it would be likely be insightful to explore whether the recreational runners change their spring-mass similarity and behavior in the same fashion as the elite runners.

### 4.6 Conclusion

We examined the systemic spring-mass similarity elite and recreational distance runners. We presented and applied several novel metrics along with a previously established method to provide a suite of measures to compare the systemic gait of runners to an ideal spring-mass system. Overall, the elite runners exhibited kinetic patterns that more closely resembled simple linearly elastic spring-mass dynamics. They demonstrated greater similarity to a symmetric bounce, and they had better coordination in their horizontal and vertical force components, indicative of more in-phase energy fluctuations within stance. Their vertical ground reaction forces correspondingly more closely fit that of a spring-mass model. These effects were generally enhanced at faster speeds within the elite runners as well as in runners exhibiting non-rearfoot striking patterns within both groups. Barefoot running elicited mixed results across measures and within groups, improving force coordination but decreasing vGRF similarity in the recreational runners with less of an effect in the elite runners. These characteristics and observations may provide further insights into the mechanical characteristics that underly superior locomotor efficiency and exceptional performance capacities. More broadly, they present new opportunities for systemic quantification of running mechanics via a canonical gait template, providing new analytical strategies to further our understanding of how runners interact with the ground.

## Data Availability

The original contributions presented in the study are included in the article and Supplementary Material. Further inquiries can be directed to the corresponding author.
